# Stability in social networks

**DOI:** 10.1098/rsos.231500

**Published:** 2024-04-24

**Authors:** Santanu Acharjee, Amlanjyoti Oza

**Affiliations:** Department of Mathematics, Gauhati University, Guwahati 781014, Assam, India

**Keywords:** social networks, Dunbar’s number, soft set, marriage, divorce, friendship

## Abstract

Dunbar’s number is the cognitive limit of human beings to maintain stable relationships with other individuals in their social networks, and it is found to be 150. It is based on the neocortex size of humans. Usually, Dunbar’s number and related phenomena are studied from the perspective of an individual. Dunbar’s number also plays a crucial role in evolutionary psychology and allied areas. However, no study done so far has considered a couple who are in a stable relationship as a system from the perspective of Dunbar’s number and its hierarchy layers. In this paper, we study the impact of Dunbar’s number and Dunbar’s hierarchy from the perspective of a couple by studying mathematically the conjoint Dunbar graphs for a couple. The cost of romance is the loss of almost two people from one’s support network when a human being enters into a new relationship. Thus, we obtain mathematically that there is no significant change in one’s friendship if human beings spend negligible time with their partners. Also, along with marriage and friendship development, we attempt to assess how a person’s social network structure holds up over the course of a romantic relationship. The stability of personal social networks is discussed through soft set theory and balance theoretic approach.

## 1. Introduction

The single most significant factor affecting our health, pleasure and well-being is friendship. Humans are social creatures, and the relationships they build with one another are essential to their growth and welfare in a society. However, the time required to establish and nurture friendships is quite expensive, as there are several cognitive processes that underlie them. In recent years, social networks have gained a lot of attention. The average size of a human’s personal social network appears to be about 150 [1]. These networks have a clearly defined layered structure with layers that are related fractally in a certain way. A human group size prediction is simple to make. In doing so, which is only a matter of extrapolating a value for group size from the primate equation using the human neocortex volume, a number in the range of 150 is obtained [2]. This value is also found in face-to-face contacts [3], calling patterns in cellphone databases [4], or postings in online environments [5], hunter–gatherer communities [6], Facebook and Twitter networks [7,8], email networks [9], co-authorship networks in scientific collaboration [7], alliances in online gaming environments [10], etc.

The variation of a personal network mainly depends on how extroverted or introverted a person is [11–14]. Compared to an introvert, an extrovert may have a few more friends, but the level of friendship depends on the size of the network of friends a person has, i.e. on average, people with a wider social network have weaker bonds [11–14]. Similar results were also observed for introverts and extroverts on many social networking sites [15,16]. A 20–30 year-old young person has a greater tendency to make friends than a 60 year-old person. This is due to the investment of time a person can afford for their friends. A 60 year-old person has less time to spend with his friends due to family obligations or other issues [3,17]. The cognitive limits on the number of persons who can be known as individuals and the constraint that time imposes on the ability for interaction both contribute to the limitations on network size and structure. Burton *et al.* [1] mentioned that these impacts are dependent on age as well, especially impacting individuals under the age of 36. The support network is even more severely diminished for people who have children. The cost of romance is the loss of nearly two members when one accounts for the addition of a new member to the network when beginning a relationship [1]. These social expenses are typically distributed evenly among network members who are related and unrelated. Dunbar [17] mentioned seven pillars of friendship, which are seven attributes that people hold in their legacies. Also, all the factors that affect the formation and decay of friendship can be considered as attributes. Since the theory of soft sets deals with attribute based approximations of objects or events [18–20], we find it the most suitable tool for discussing the whole scenario using soft sets. We have made a detailed analysis of these events, such as friendship, stability of friendship, marriage and divorce in the later sections. We focus mainly on the following questions:

(i)
How do the Dunbar’s layers of friendship work when a person gets married, i.e. the study of the Dunbar graph for a couple?
(ii)
How can we measure the bonding of friendship between two people mathematically?
(iii)
How does time investment affect the relationship between friendship and romance?
(iv)
How do personal social networks develop, and how big can a network get before it starts to become unstable due to too many weak links, given the distribution of the seven pillar qualities in the population?
(v)
What is the likelihood of a married couple having extramarital affairs?

## 2. Soft sets and conjoint Dunbar graphs

We cannot effectively handle complex problems in social science, economics and the environment with classical methods due to a number of inherent uncertainties. However, a potentially useful general mathematical tool for handling ambiguous, fuzzily specified things is provided by soft set theory. In this paper, we will look at how numerous social elements affect people’s connections with one another. The theory of soft sets, which deals with the parameters that influence the approximations of an attribute through mapping, offers a practical solution for this. Thus, we consider the concept of soft set theory to study the relationship between friendship and romantic relationships and the stability of one’s personal social network with related events.

**Definition 2.1. ([18])**

A pair (*F*, *A*) is called a soft set over the universal set *X* if and only if *F* is a mapping of A into the set of all subsets of the set X, i.e. *F* : *A* → 2^*X*^. In other words, the soft set is a parametrized family of subsets of the set *X*.

Since parametrization is an auxiliary factor for the convenience of working with soft sets, it is natural to introduce the notion of equivalence of soft sets. If the soft set (*F*, *A*) is given, then the family *τ*(*F*, *A*) = {*F*(*a*) : *a* ∈ *A*} specifies those subsets that can be approximate descriptions, and the parameter set *A* is chosen for reasons of convenience by the person who introduces the definition of this soft set.

**Example 2.2.**

Let us consider a universal set *X* = {*a*, *b*, *c*, *d*} of four houses and a set of three attributes, say *A* = {*e*_1_, *e*_2_, *e*_3_}. We define a mapping $F\;:\;A⟶2^{X}$ such that *F*(*e*_1_) = {*a*, *b*}, *F*(*e*_2_) = {*b*, *c*, *d*}, and *F*(*e*_3_) = {*a*, *c*, *d*}. Then, the soft set (*F*, *A*) is defined as (*F*, *A*) = {(*e*_1_, {*a*, *b*}), (*e*_2_, {*b*, *c*, *d*}), (*e*_3_, {*a*, *c*, *d*})}. Thus, *τ*(*F*, *A*) = {{*a*, *b*}, {*b*, *c*, *d*}, {*a*, *c*, *d*}}.

We procure some fundamental operations of soft sets below:

**Definition 2.3. ([21])**

Two soft sets (*F*, *A*) and (*F*′, *A*′) given over the universal set *X* will be called equal, and we write (*F*, *A*) = (*F*′, *A*′) if and only if *F* = *F*′ and *A* = *A*′.

**Definition 2.4. ([21])**

Two soft sets (*F*, *A*) and (*F*′, *A*′) given over the universal set *X* will be called equivalent and written as (*F*, *A*) ≅ (*F*′, *A*′) if and only if *τ*(*F*, *A*) = *τ*(*F*′, *A*′).

It is easy to note that the equivalence of soft sets is an equivalence relation.

**Definition 2.5. ([21])**

The unary operation complement of (*F*, *A*) over a universal set *X*, denoted by *C*(*F*, *A*) = (*W*, *A*), is defined as follows. The set of parameters remains the same, and the mapping is given by W(a)=X∖F(a) for any *a* ∈ *A*.

**Definition 2.6. ([21])**

The binary operation union (S,A)∪(F,D)=(H,A×D) for a pair of soft sets (*S*, *A*) and (*F*, *D*) given over a universal set *X* is defined as follows: The parameter set is chosen equal to the direct product of the parameter sets, i.e. equal to *A* × *D*, and the corresponding mappings are given by the formula H(a,d)=S(a)∪F(d) , (*a*, *d*) ∈ *A* × *D*.

**Definition 2.7. ([21])**

The binary operation intersection (S,A)∩(F,D)=(W,A×D) for a pair of soft sets (*S*, *A*) and (*F*, *D*) given over a universal set *X* is defined as follows: The parameter set is chosen equal to the direct product of the parameter sets, i.e. equal to *A* × *D*, and the corresponding mappings are given by the formula W(a,d)=S(a)∩F(d) , (*a*, *d*) ∈ *A* × *D*.

**Definition 2.8. ([22])**

The binary operation product (*S*, *A*) × (*F*, *D*) = (*X*, *A* × *D*) for a pair of soft sets (*S*, *A*) and (*F*, *D*) given over a universal set *X* is defined as follows. The parameter set is chosen equal to the direct product of the parameter sets, i.e. equal to *A* × *D* and the corresponding mappings are given by the formula *X*(*a*, *d*) = *S*(*a*) × *F*(*d*), (*a*, *d*) ∈ *A* × *D*.

Now, we consider an illustrative example to discuss the above operations.

**Example 2.9.**

Let us construct two soft sets first. Consider the mappings $F\;:\;A⟶2^{X}$ and $G\;:\;B⟶2^{X}$ for two soft sets (*F*, *A*) and (*G*, *B*) over a universal set *X*. Let *X* contain people from a particular place, and let *A*, *B* be two sets of attributes. Consider *X* = {*a*, *b*, *c*, *d*, *e*}, A={tall, short} , and B={girl, boy} . *F*(tall) = {*a*, *b*, *c*, *d*}, *F*(short) = {*e*}. Therefore, the soft (*F*, *A*) = {(tall, {*a*, *b*, *c*, *d*}), (short, {*e*})}. Hence, *τ*(*F*, *A*) = {{*a*, *b*, *c*, *d*}, {*e*},}. Similarly, *G*(girl) = {*a*, *b*, *c*}, *G*(boy) = {*d*, *e*}. Therefore, the soft set (*G*, *B*) = {(girl, {*a*, *b*, *c*}), (boy, {*d*, *e*})}. Hence, *τ*(*G*, *B*) = {{*a*, *b*, *c*}, {*d*, *e*}}.

Now, we calculate the unary operation complement ‘*C*’ of the soft set (*F*, *A*) which is given by *C*(*F*, *A*), where C(a)=X∖F(a),∀a∈A . So, *C*(tall)={*e*}, and *C*(short)={*a*, *b*, *c*, *d*}. Therefore, *C*(*F*, *A*) = {(tall, {*e*}), (short, {*a*, *b*, *c*, *d*})}. Thus, *τ*(*C*, *A*) = {{*e*}, {*a*, *b*, *c*, *d*}}.

Again, we consider the binary operation union ‘ ⋃ ’ between the soft sets (*F*, *A*) and (*G*, *B*) denoted by (F,A)⋃(G,B)=(H,A×B) , where *A* × *B* is the Cartesian product of *A* and *B*, and the mapping is given by H(a,b)=F(a)∪G(a) , where (*a*, *b*) ∈ *A* × *B*. Thus, *A* × *B* = {{tall, girl}, {tall, boy}, {short, girl}, {short, boy}}. So, we obtain *H*(tall, girl)={*a*, *b*, *c*, *d*}, *H*(tall, boy) = {*a*, *b*, *c*, *d*, *e*}, *H*(short, girl) = {*a*, *b*, *c*, *e*}, and *H*(short, boy) = {*d*, *e*}. Hence, (*H*, *A* × *B*) = {((tall, girl), {*a*, *b*, *c*, *d*}), ((tall, boy), {*a*, *b*, *c*, *d*, *e*}), ((short, girl), {*a*, *b*, *c*, *e*}), ((short, boy), {*d*, *e*})}. Therefore, *τ*(*H*, *A* × *B*) = {{*a*, *b*, *c*, *d*}, {*a*, *b*, *c*, *d*, *e*}, {*a*, *b*, *c*, *e*}, {*d*, *e*}}.

Now, we also consider the binary operation intersection ‘ ⋂ ’ between (*F*, *A*) and (*G*, *B*) denoted by (F,A)⋂(G,B)=(W,A×B) , and the mapping is given by W(a,b)=F(a)∩G(b) , where (*a*, *b*) ∈ *A* × *B*. So, we obtain *W*(tall, girl) = {*a*, *b*, *c*}, *W*(tall, boy) = {*d*}, W(short,girl)=∅ and *W*(short, boy)={*e*}. Therefore, (W,A×B)={((tall,girl),{a,b,c}),((tall, boy),{d}),((short,girl),∅),((short,boy),{e})} . Hence, *τ*(*W*, *A* × *B*) = {{*a*, *b*, *c*}, {*d*}, {*e*}}.

Also, we consider the binary operation product ‘×’ between (*F*, *A*) and (*G*, *B*), denoted by (*F*, *A*) × (*G*, *B*) = (*X*, *A* × *B*), and the mapping is given by *X*(*a*, *b*) = *F*(*a*) × *G*(*b*), where (*a*, *b*) ∈ *A* × *B*. So, we obtain, *X*(tall, girl)={*a*, *b*, *c*, *d*} × {*a*, *b*, *c*} = {(*a*, *a*), (*a*, *b*), (*a*, *c*), (*b*, *a*), (*b*, *b*), (*b*, *c*), (*c*, *a*), (*c*, *b*), (*c*, *c*), (*d*, *a*), (*d*, *b*), (*d*, *c*)}, *X*(tall, boy) = {*a*, *b*, *c*, *d*} × {*d*, *e*} = {(*a*, *d*), (*a*, *e*), (*b*, *d*), (*b*, *e*), (*c*, *d*), (*c*, *e*), (*d*, *d*), (*d*, *e*)}, *X*(short, girl) = {*e*} × {*a*, *b*, *c*} = {(*e*, *a*), (*e*, *b*), (*e*, *c*)}, and *X*(short, boy) = {*e*} × {*d*, *e*} = {(*e*, *d*), (*e*, *e*)}. Therefore, (*X*, *A* × *B*) = {((tall, girl), {(*a*, *a*), (*a*, *b*), (*a*, *c*), (*b*, *a*), (*b*, *b*), (*b*, *c*), (*c*, *a*), (*c*, *b*), (*c*, *c*), (*d*, *a*), (*d*, *b*), (*d*, *c*)}), ((tall, boy), {(*a*, *d*), (*a*, *e*), (*b*, *d*), (*b*, *e*), (*c*, *d*), (*c*, *e*), (*d*, *d*), (*d*, *e*)}), ((short, girl), {(*e*, *a*), (*e*, *b*), (*e*, *c*)}), ((short, boy), {(*e*, *d*), (*e*, *e*)})}. Hence, *τ*(*X*, *A* × *B*) = {(*a*, *a*), (*a*, *b*), (*a*, *c*), (*b*, *a*), (*b*, *b*), (*b*, *c*), (*c*, *a*), (*c*, *b*), (*c*, *c*), (*d*, *a*), (*d*, *b*), (*d*, *c*)}, {(*a*, *d*), (*a*, *e*), (*b*, *d*), (*b*, *e*), (*c*, *d*), (*c*, *e*), (*d*, *d*), (*d*, *e*)}, {(*e*, *a*), (*e*, *b*), (*e*, *c*)}, {(*e*, *d*), (*e*, *e*)}.

For more on soft set theory, one may refer to [18,21,22].

**Figure 1.**
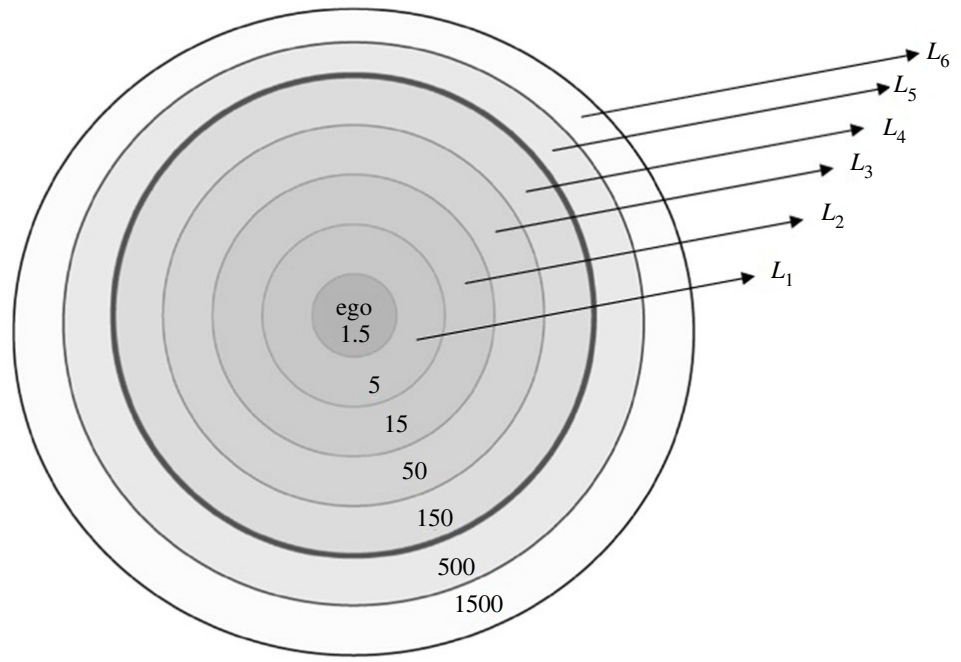
Dunbar’s hierarchy of social structure.

The hierarchy of friendship, as shown in figure 1, depicts the friends of a person depending on emotional closeness. The innermost layer of an ego consists of 1.5 people, as obtained by regression analysis [23], within hierarchically inclusive friendship circles or layers that have a very specific scaling ratio, i.e. each layer is three times the size of the one inside it. Hence, we are considering the layers starting from *L*_1_ during the formation of friendships. Similarly, later on, in the case of the analysis of social networks formation with respect to a couple, we take the couple as an ego. Layer *L*_1_ is made up of the person’s closest acquaintances and is also referred to as their support system. Compared to one’s acquaintances from layer *L*_1_, those in layer *L*_2_ are those with whom the person has less emotional intimacy. In a similar manner, we obtain the other layers *L*_3_, *L*_4_, *L*_5_ and *L*_6_ etc. of Dunbar’s friendship hierarchy. They denote the degree of a person’s friendships with their peers in each circle. The 150 layer, which is the typical size for personal social networks, is denoted by the thick line. The layer at 1500 appears to reflect the average number of faces we are able to assign names to. There are acquaintances (500 layer) after this layer. Each succeeding circle sees a drop in contact frequency, assessed emotional intimacy, and readiness to act benevolently towards a particular alter. Next to the innermost layer, there are five people who are very closely emotionally connected to the person. This circle generally consists of immediate close family, best friends, or a romantic partner as well [17]. As shown in the figure, as we go outward from the centre of the circle, the number of people increases but the emotional closeness decreases gradually. In this section, we try to connect two persons’ friendship circles so that if the two persons get married, then how many friends they may have in common within a specific layer of the hierarchy of friendship is determined through some notions of soft set theory. For this, we define the following definitions:

We first consider the operations for two soft sets (*F*, *A*) and (*G*, *B*) defined on the universal set X. The relationship between the parameters *a* ∈ *A* and *b* ∈ *B* will be described as an ordered pair (*a*, *b*). All ordered pairs form the set Con⊆ *A* × *B*. Denote *π*(Con, *a*,.) = {*b* : (*a*, *b*) ∈ Con}; and *π*(Con,., *b*) = {*a* : (*a*, *b*) ∈ Con}.

**Definition 2.10.**

(i) A left narrow union of soft sets (*F*, *A*) and (*G*, *B*) with the set Con⊆ *A* × *B* is the soft set (*H*, *A*), where
$$H(a)=\left\{ \begin{array}{cc} F(a)\cup \{\cap_{b\in \pi (\mathrm{Con},a,.)}G(b)\}\; & \mathrm{if}\;\pi (\mathrm{Con},a,.)\neq \emptyset ,\;\\ F(a)\; & \mathrm{if}\;\pi (\mathrm{Con},a,.)=\emptyset .\;\; \end{array}\right.$$
  It is denoted by $\left(H,A)=(F,A)\cup_{\mathrm{LCon}}^{-}(G,B\right)$ .
(ii)
A left wide union of soft sets (*F*, *A*) and (*G*, *B*) with the set Con⊆ *A* × *B* is the soft set (*H*, *A*), where
$$H(a)=\left\{ \begin{array}{cc} F(a)\cup \{\cup_{b\in \pi (\mathrm{Con},a,.)}G(b)\}\; & \mathrm{if}\;\pi (\mathrm{Con},a,.)\neq \emptyset ,\;\\ F(a)\; & \mathrm{if}\;\pi (\mathrm{Con},a,.)=\emptyset .\;\\ \; \end{array}\right.$$
  It is denoted by $\left(H,A)=(F,A)\cup_{\mathrm{LCon}}^{+}(G,B\right)$ .
(iii)
A left narrow intersection of soft sets (*F*, *A*) and (*G*, *B*) with the set Con⊆ *A* × *B* is the soft set (*H*, *A*), where
$$H(a)=\left\{ \begin{array}{cc} F(a)\cap \{\cap_{b\in \pi (\mathrm{Con},a,.)}G(b)\}\; & \mathrm{if}\;\pi (\mathrm{Con},a,.)\neq \emptyset ,\;\\ F(a)\; & \mathrm{if}\;\pi (\mathrm{Con},a,.)=\emptyset .\;\\ \; \end{array}\right.$$
  It is denoted by $\left(H,A)=(F,A)\cap_{\mathrm{LCon}}^{-}(G,B\right)$ .
(iv)
A left wide intersection of soft sets (*F*, *A*) and (*G*, *B*) with the set Con⊆ *A* × *B* is the soft set (*H*, *A*), where
$$H(a)=\left\{ \begin{array}{cc} F(a)\cap \{\cup_{b\in \pi (\mathrm{Con},a,.)}G(b)\}\; & \mathrm{if}\;\pi (\mathrm{Con},a,.)\neq \emptyset ,\;\\ F(a)\; & \mathrm{if}\;\pi (\mathrm{Con},a,.)=\emptyset .\;\\ \; \end{array}\right.$$
  It is denoted by $\left(H,A)=(F,A)\cap_{\mathrm{LCon}}^{+}(G,B\right)$ .

Now, let us analyse the situations mentioned above. We define soft sets (*F*, *A*) and (*G*, *B*) for two individuals *X* and *Y*, respectively, before they get into a relationship with each other. We consider the universal set *U* consisting of four main layers of Dunbar’s hierarchy, i.e. *U* = {*L*_1_, *L*_2_, *L*_3_, *L*_4_}. Also, we consider the sets of attributes *A* and *B* consisting of the friends of *X* and *Y*, respectively, in Dunbar’s hierarchy layers not exceeding Dunbar’s number 150. For the sake of ease in our current analysis, we consider *F*(*a*_1_) and *F*(*a*_2_) as approximations of *a*_1_ and *a*_2_ ∈ *A* for the soft set (*F*, *A*). Similarly, *G* (*b*_1_), *G*(*b*_2_) and *G*(*b*_3_) are approximations of *b*_1_, *b*_2_ and *b*_3_ ∈ *B* for the soft set (*G*, *B*). Now, if *a*_1_ lies inside layer *L*_3_ of Dunbar’s hierarchy layers, then obviously *a*_1_ will be inside layer *L*_4_. So, we assume that *F*(*a*_1_) = {*L*_3_, *L*_4_}, and *F*(*a*_2_) = {*L*_2_, *L*_3_, *L*_4_}. Similarly, we consider *G*(*b*_1_) = {*L*_1_, *L*_2_, *L*_3_, *L*_4_}, *G*(*b*_2_) = {*L*_2_, *L*_3_, *L*_4_}, and *G*(*b*_3_) = {*L*_4_}. Now, imagine the situation when *X* marries *Y* and they become a couple C . Thus, we have to analyse the scenario of Dunbar’s hierarchy layers for this couple. We define the set Con = {(*a*, *b*) : *a* is interested in being a friend of *b*}⊆ *A* × *B*. If *a*_1_ is interested in being a friend of *b*_1_, *b*_2_ and *b*_3_, and *a*_2_ is interested in being a friend of only *b*_1_ and *b*_2_ then Con = {(*a*_1_, *b*_1_), (*a*_1_, *b*_2_), (*a*_1_, *b*_3_), (*a*_2_, *b*_1_), (*a*_2_, *b*_2_)}. Thus, we get table 1.

**Table 1.** Position of the individuals *a*_1_ and *a*_2_ in four layers Dunbar’s hierarchy of the couple C from the perspective of the individual *X*.

(*H*, *A*)=	$\left(F,A)\cup_{\mathrm{LCon}}^{-}(G,B\right)$	$\left(F,A)\cup_{\mathrm{LCon}}^{+}(G,B\right)$	$\left(F,A)\cap_{\mathrm{LCon}}^{-}(G,B\right)$	$\left(F,A)\cap_{\mathrm{LCon}}^{+}(G,B\right)$
*H*(*a*_1_)=	{*L*_3_, *L*_4_}	{*L*_1_, *L*_2_, *L*_3_, *L*_4_}	{*L*_4_}	{*L*_3_, *L*_4_}
*H*(*a*_2_) =	{*L*_2_, *L*_3_, *L*_4_}	{*L*_1_, *L*_2_, *L*_3_, *L*_4_}	{*L*_2_, *L*_3_, *L*_4_}	{*L*_2_, *L*_3_, *L*_4_}

From this example, along with the definitions of various types of unions and intersections between two soft sets as defined above, we conclude the following:

In the cases of left narrow union and left narrow intersection, we calculate the expression $\left\{\cap_{b\in \pi (\mathrm{Con},a,.)}G(b)\right\}$ , if π(Con,a,.)≠∅ , which gives us the layers in Dunbar’s hierarchy that consist of the people with whom Y has less emotional closeness than the others. After that, the left narrow union $F(a_{1})\cup \{\cap_{b\in \pi (\mathrm{Con},a,.)}G(b)\}$ indicates the layers of Dunbar’s friendship hierarchy for the couple C in which either *X* or *Y* decides to keep *a*_1_ and the persons that lie in the layers we get while taking the intersection $\left\{\cap_{b\in \pi (\mathrm{Con},a,.)}G(b)\right\}$ . On the other hand, when we take the left narrow intersection $F(a_{1})\cap \{\cap_{b\in \pi (\mathrm{Con},a,.)}G(b)\}$ , it indicates the layers of Dunbar’s hierarchy for C in which both *X* and *Y* decide to keep the person *a*_1_ and the persons that lie in the layers we get while taking the intersection $\left\{\cap_{b\in \pi (\mathrm{Con},a,.)}G(b)\right\}$ .

In the cases of left wide union and left wide intersection, we calculate the expression

$\left\{\cup_{b\in \pi (\mathrm{Con},a,.)}G(b)\right\}$ , if π(Con,a,.)≠∅ . It gives us the layers in the Dunbar hierarchy that consist of the people with whom Y has better emotional closeness than the others. After that, the left wide union $F(a_{1})\cup \{\cup_{b\in \pi (\mathrm{Con},a,.)}G(b)\}$ indicates the layers of Dunbar’s hierarchy for the couple C in which either *X* or *Y* decides to keep *a*_1_ and the persons that lie in the layers we get while taking the union $\left\{\cup_{b\in \pi (\mathrm{Con},a,.)}G(b)\right\}$ . On the other hand, when we take the left wide intersection $F(a_{1})\cap \{\cup_{b\in \pi (\mathrm{Con},a,.)}G(b)\}$ , it indicates the layers of Dunbar’s hierarchy for C in which both *X* and *Y* decide to keep the person *a*_1_ and the persons that lie in the layers we get while taking the union $\left\{\cup_{b\in \pi (\mathrm{Con},a,.)}G(b)\right\}$ . From the viewpoint of *X*, we have seen the friendship hierarchy of a couple C of two people *X* and *Y*. The following definitions can be taken into account if we attempt to assess the identical circumstance from the viewpoint of *Y*:

**Definition 2.11.**

(i)
A right narrow union of soft sets (*F*, *A*) and (*G*, *B*) with the set Con⊆ *A* × *B* is the soft set (*H*, *B*), where
$$H(b)=\left\{ \begin{array}{cc} \{\cap_{a\in \pi (\mathrm{Con},.,b)}F(a)\}\cup G(b)\; & \mathrm{if}\;\pi (\mathrm{Con},.,b)\neq \emptyset ,\;\\ G(b)\; & \mathrm{if}\;\pi (\mathrm{Con},.,b)=\emptyset .\;\; \end{array}\right.$$
  It is denoted by $\left(H,B)=(F,A)\cup_{\mathrm{RCon}}^{-}(G,B\right)$ .
(ii)
A right wide union of soft sets (*F*, *A*) and (*G*, *B*) with the set Con⊆ *A* × *B* is the soft set (*H*, *B*), where
$$H(b)=\left\{ \begin{array}{cc} \{\cup_{a\in \pi (\mathrm{Con},.,b)}F(a)\}\cup G(b)\; & \mathrm{if}\;\pi (\mathrm{Con},.,b)\neq \emptyset ,\;\\ G(b)\; & \mathrm{if}\;\pi (\mathrm{Con},.,b)=\emptyset .\;\; \end{array}\right.$$
  It is denoted by $\left(H,B)=(F,A)\cup_{\mathrm{RCon}}^{+}(G,B\right)$ .
(iii)
A right narrow intersection of soft sets (*F*, *A*) and (*G*, *B*) with the set Con⊆ *A* × *B* is the soft set (*H*, *B*), where
$$H(b)=\left\{ \begin{array}{cc} \{\cap_{a\in \pi (\mathrm{Con},.,b)}F(a)\}\cap G(b)\; & \mathrm{if}\;\pi (\mathrm{Con},.,b)\neq \emptyset ,\;\\ G(b)\; & \mathrm{if}\;\pi (\mathrm{Con},.,b)=\emptyset .\;\; \end{array}\right.$$
  It is denoted by $\left(H,B)=(F,A)\cap_{\mathrm{RCon}}^{-}(G,B\right)$ .
(iv)
A right wide intersection of soft sets (*F*, *A*) and (*G*, *B*) with the set Con⊆ *A* × *B* is the soft set (*H*, *B*), where
$$H(b)=\left\{ \begin{array}{cc} \{\cup_{a\in \pi (\mathrm{Con},.,b)}F(a)\}\cap G(b)\; & \mathrm{if}\;\pi (\mathrm{Con},.,b)\neq \emptyset ,\;\\ G(b)\; & \mathrm{if}\;\pi (\mathrm{Con},.,b)=\emptyset .\;\; \end{array}\right.$$
  It is denoted by $\left(H,B)=(F,A)\cap_{\mathrm{RCon}}^{+}(G,B\right)$ .

From the above definitions, we see that the basic difference between left narrow union/intersection and right narrow union/intersection is that, for left narrow union/intersection we consider all the attributes from the soft set (*F*, *A*) and only those attributes of the soft set (*G*, *B*) which are connected to *a* ∈ *A* through the set Con and for right narrow union/intersection, we consider all the attributes from the soft set (*G*, *B*) and only those attributes of the soft set (*F*, *A*) that are connected to *b* ∈ *B* through the set Con. Similarly, for left wide union/intersection we consider all the attributes from the soft set (*F*, *A*) and only those attributes of the soft set (*G*, *B*) that are connected to *a* ∈ *A* through the set Con and for right wide union/intersection, we consider all the attributes from the soft set (*G*, *B*) and only those attributes of the soft set (*F*, *A*) that are connected to *b* ∈ *B* through the set Con. These scenarios are quite natural in the case of couple formation.

**Definition 2.12.**

(i)
The symmetric union of (*F*, *A*) and (*G*, *B*) with the set Con⊆ *A* × *B* is the soft set (*H*, Con), where H(a,b)=F(a)∪G(b) . We denote it as $\left(H,\mathrm{Con})=(F,A)\cup_{\mathrm{Con}}(G,B\right)$ .
(ii)
The symmetric intersection of (*F*, *A*) and (*G*, *B*) with the set Con⊆ *A* × *B* is the soft set (*H*′, Con), where $H^{\prime }(a,b)=F(a)\cap G(b)$ . We denote it as $\left(H^{\prime },\mathrm{Con})=(F,A)\cap_{\mathrm{Con}}(G,B\right)$ .

One of the most important properties of classical union and intersection is commutativity. So, for a narrow union we have to compare two soft sets $\left(F,A)\cup_{\mathrm{Con}}^{-}(G,B\right)$ and $\left(G,B)\cup_{{\mathrm{Con}}^{{\;}^{\prime }}}^{-}(F,A\right)$ , where Con^′^ = {(*b*, *a*) ∈ *B* × *A* : (*a*, *b*) ∈ Con}. Naturally, there is no point in talking about the equality of these soft sets. It only makes sense to find out whether they are equivalent. Unfortunately, for operations: narrow union, wide union, narrow intersection, wide intersection, this property, generally speaking, is not fair.

**Theorem 2.13.**

*Suppose that the following conditions are satisfied for two soft sets* (*F*, *A*) *and* (*G*, *B*) *over a universe* *X* *and the set* *Con⊆* *A* × *B*.

1.
*For every* *a* ∈ *A*; *the set* *π*(*Con*, *a*,.) *is a singleton set*,
2.
*For every* *b* ∈ *B*; *the set* *π*(*Con*,., *b*) *is a singleton set*.

*Then*,

(i)
$\left(F,A)\cap_{LCon}^{-}(G,B)=(F,A)\cap_{LCon}^{+}(G,B\right)$ ,
(ii)
$\left(F,A)\cup_{LCon}^{-}(G,B)=(F,A)\cup_{LCon}^{+}(G,B\right)$ ,
(iii)
$\left(F,A)\cup_{LCon}^{-}(G,B)=(G,B)\cup_{LCon}^{-}(F,A\right)$ ,
(iv)
$\left(F,A)\cap_{LCon}^{-}(G,B)=(G,B)\cap_{LCon}^{-}(F,A\right)$ ,
(v)
$\left(F,A)\cup_{LCon}^{+}(G,B)=(G,B)\cup_{LCon}^{+}(F,A\right)$ ,
(vi)
$\left(F,A)\cap_{LCon}^{+}(G,B)=(G,B)\cap_{LCon}^{+}(F,A\right)$ .

**Proof.**

We shall prove only (*i*), (*iii*) and (*v*). Since *π*(Con, *a*,.) and *π*(Con,., *b*) are singleton sets, let *π*(Con, *a*,.) = {*b*} and *π*(Con,., *b*) = {*a*}.

(i)
For $\left(F,A)\cap_{\mathrm{LCon}}^{-}(G,B\right)$ , $F(a)\cap \{\cap_{b\in \pi (\mathrm{Con},a,.)}G(b)\}=F(a)\cap G(b)$ . Also for $\left(F,A)\cap_{\mathrm{LCon}}^{+}(G,B\right)$ , $F(a)\cap \{\cup_{b\in \pi (\mathrm{Con},a,.)}G(b)\}=F(a)\cap G(b)$ , for all *a* ∈ *A* and *b* ∈ *B*. Thus, $\left(F,A)\cap_{\mathrm{LCon}}^{-}(G,B)=(F,A)\cap_{\mathrm{LCon}}^{+}(G,B\right)$ .
(ii)
For $\left(F,A)\cup_{\mathrm{LCon}}^{-}(G,B\right)$ , $F(a)\cup \{\cap_{b\in \pi (\mathrm{Con},a,.)}G(b)\}=F(a)\cup G(b)$ . Also for $\left(G,B)\cup_{\mathrm{LCon}}^{-}(F,A\right)$ , $G(b)\cup \{\cap_{a\in \pi (\mathrm{Con},.,b)}F(a)\}=G(b)\cup F(a)=F(a)\cup G(b)$ , for all *a* ∈ *A* and *b* ∈ *B*. Thus, $\left(F,A)\cup_{\mathrm{LCon}}^{-}(G,B)=(G,B)\cup_{\mathrm{Con}}^{-}(F,A\right)$ .
(iii)
For $\left(F,A)\cup_{\mathrm{LCon}}^{+}(G,B\right)$ , $F(a)\cup \{\cup_{b\in \pi (\mathrm{Con},a,.)}G(b)\}=F(a)\cup G(b)$ . Also for $\left(G,B)\cup_{\mathrm{LCon}}^{+}(F,A\right)$ , $G(b)\cup \{\cup_{a\in \pi (\mathrm{Con},.,b)}F(a)\}=G(b)\cup F(a)=F(a)\cup G(b)$ , for all *a* ∈ *A* and *b* ∈ *B*. Thus, $\left(F,A)\cup_{\mathrm{LCon}}^{+}(G,B)=(G,B)\cup_{\mathrm{LCon}}^{+}(F,A\right)$ .

■

**Theorem 2.14.**

*Suppose that the following conditions are satisfied for two soft sets* (*F*, *A*) *and* (*G*, *B*) *over a universe* *X* *and the set* *Con⊆* *A* × *B*.

1.
*For every* *a* ∈ *A*; *the set* *π*(*Con*, *a*,.) *is a singleton set*,
2.
*For every* *b* ∈ *B*; *the set* *π*(*Con*,., *b*) *is a singleton set*.

*Then*,

(i)
$\left(F,A)\cap_{RCon}^{-}(G,B)=(F,A)\cap_{RCon}^{+}(G,B\right)$ ,
(ii)
$\left(F,A)\cup_{RCon}^{-}(G,B)=(F,A)\cup_{RCon}^{+}(G,B\right)$ ,
(iii)
$\left(F,A)\cup_{RCon}^{-}(G,B)=(G,B)\cup_{RCon}^{-}(F,A\right)$ ,
(iv)
$\left(F,A)\cap_{RCon}^{-}(G,B)=(G,B)\cap_{RCon}^{-}(F,A\right)$ ,
(v)
$\left(F,A)\cup_{RCon}^{+}(G,B)=(G,B)\cup_{RCon}^{+}(F,A\right)$

(vi)
$\left(F,A)\cap_{RCon}^{+}(G,B)=(G,B)\cap_{RCon}^{+}(F,A\right)$ .

**Proof.**

Similar to the proofs of the previous theorem.■

Now, let us consider two soft sets (*F*, *A*) and (*G*, *B*), respectively, for two persons with the parameter sets equal to the seven pillars of friendship [17], i.e. *A* = *B* ={ language, place of origin, educational history, hobbies/interests, sense of humour, worldview, musical tastes} and the universal set *X* is equal to the attribute values of the elements of *A* or *B*, i.e. the languages all over the world, the places all over the world, and so on. Here, we shall take the set $\mathrm{Con}=\{(a_{i},a_{i})\;:\;(a_{i},a_{i})\in A\times B,\forall a_{i}\in A\;\mathrm{or}\;B,i=1,2,\ldots ,7\}\subseteq A\times B$ . In this case, for every *a*_*i*_ ∈ *A* or *B*, *i* = 1, 2, …, 7, the set *π*(Con, *a*_*i*_,.) is a singleton set. Then, we find the soft sets $\left(F,A)\cap_{\mathrm{LCon}}^{-}(G,B\right)$ or $\left(F,A)\cap_{\mathrm{LCon}}^{+}(G,B\right)$ to know how much is common between the two people under consideration. The higher cardinality of the sets $\tau ((F,A)\cap_{\mathrm{LCon}}^{-}(G,B))$ or $\tau ((F,A)\cap_{\mathrm{LCon}}^{+}(G,B))$ will indicate a higher possibility of the two people to become friends. Let us take the example below:

**Example 2.15.**

We take minimum number of attribute values for the universal set *X* so that the calculations get easier. We consider five languages: $L_{1},L_{2},\ldots ,L_{5}$ , five places around the world: $P_{1},P_{2},\ldots ,P_{5}$ , five educational institutions: $E_{1},E_{2},\ldots ,E_{5}$ , five hobbies: $H_{1},H_{2},\ldots ,H_{5}$ , two senses of humour: *S*_1_ and *S*_2_, two political views: *V*_1_, *V*_2_, five musical tastes: $M_{1},M_{2},\ldots ,M_{5}$ for generating the universal set *X*. Now we define the soft set (*F*, *A*) as follows: *F*(language) = {*L*_2_, *L*_5_}, *F*(place of origin) = {*P*_3_}, *F*(educational history) = {*E*_3_, *E*_4_, *E*_5_}, *F*(hobby) = {*H*_1_, *H*_3_, *H*_5_}, *F*(sense of humour) = {*S*_2_}, *F*(political view) = {*V*_2_} and *F*(musical taste) = {*M*_1_, *M*_2_, *M*_3_}. Similarly, we define another soft set (*G*, *B*) as follows: *G*(language) = {*L*_2_, *L*_4_}, *G*(place of origin) = {*P*_3_}, *G*(educational history) = {*E*_3_, *E*_2_}, *G*(hobby) = {*H*_1_, *H*_3_, *H*_5_}, *G*(sense of humour) = {*S*_1_}, *G*(political view) = {*V*_2_} and *G*(musical taste) = {*M*_1_, *M*_3_, *M*_4_}. Now, we construct the set Con as, Con ={(language, language), (place of origin, place of origin), (educational history, educational history), (hobby, hobby), (sense of humour, sense of humour), (moral view, moral view), (musical taste, musical taste)}. We calculate the sets $\tau ((F,A)\cap_{\mathrm{LCon}}^{-}(G,B))=\{\{L_{2}\},\{P_{3}\},\{E_{3}\},\{H_{1},H_{3},H_{5}\},\{V_{2}\},\{M_{1},M_{3}\}\}$ and $\tau ((F,A)\cap_{\mathrm{LCon}}^{+}(G,B))=\{\{L_{2}\},\{P_{3}\}$ , {*E*_3_}, {*H*_1_, *H*_3_, *H*_5_}, {*V*_2_}, {*M*_1_, *M*_3_}}. Here, higher cardinalities of the sets $\tau ((F,A)\cap_{\mathrm{LCon}}^{-}(G,B))$ or $\tau ((F,A)\cap_{\mathrm{LCon}}^{+}(G,B))$ will indicate higher possibilities of creating a relationship between the two people under consideration.

**Theorem 2.16.**

*For any two soft sets* (*F*, *A*) *and* (*G*, *B*) *over a universe* *X* *and the set* *Con⊆* *A* × *B*, *the following conditions hold:*

(i)
$\left(F,A)\cup_{Con}(G,B)=(G,B)\cup_{{Con}^{{\;}^{\prime }}}(F,A\right)$

(ii)
$\left(F,A)\cap_{Con}(G,B)=(G,B)\cap_{{Con}^{{\;}^{\prime }}}(F,A\right)$

**Proof.**

We only give the proof of (i).

Let $\left(H,\mathrm{Con})=(F,A)\cup_{\mathrm{Con}}(G,B\right)$ . Then, the mapping *H* is given by H(a,b)=F(a)∪G(b) , where Con⊆ *A* × *B*. Also, if $\left(H^{\prime },{\mathrm{Con}}^{\prime })=(G,B)\cup_{\mathrm{Con}}(F,A\right)$ , then the mapping *H*′ is given by $H^{\prime }(b,a)=G(b)\cup F(a)=F(a)\cup G(b)$ , where Con′⊆ *B* × *A*. Thus, $\left(F,A)\cup_{\mathrm{Con}}(G,B)=(G,B)\cup_{{\mathrm{Con}}^{{\;}^{\prime }}}(F,A\right)$ .■

Here, we should note that the sets of attributes *A* × *B* and *B* × *A* are indistinguishable while calculating the union and intersection of two soft sets having the parameter sets *A* and *B*, respectively [22].

## 3. Analysis of the formation of friendships and their effect on romantic relationships

In [17], Dunbar noted that friendship is the most important factor for our happiness, mental well-being, and health. He defined friends as the people who share our lives in a way that is more than just the casual meeting of strangers. It may include friends, members of our extended family and our romantic partners. Emotional closeness is defined as how an individual feels about another and can be determined by a variety of psychological instruments. Here, we try to find the emotional closeness or deepness of friendship between two individuals through some mathematical tools.

Dunbar [17] demonstrated how people who are friends often have a lot in common. Personal social networks are frequently homophilous by gender; for instance, men’s networks tend to contain a disproportionate number of men, and women’s networks tend to contain a disproportionate number of women [12]. McPherson *et al.* [24] also identified several factors in homophilous networks, viz., gender, ethnicity, age, religion, education, social values, etc. Similarly, Newmann [25,26] studied assortative mixing in networks from the perspective of factors like language and race. Assortative mixing is the tendency for vertices in networks to be connected to other vertices due to homophily. He also found strong variation with assortativity in the connectivity of the networks. Dunbar [17] identifies seven dimensions as the foundation of friendship: language, place of origin, career trajectory, hobbies/interests, sense of humour, worldview, and musical tastes. He also mentioned that these seven pillars seem to be interchangeable, and a four-star relationship can involve any combination of these seven dimensions [17]. From the above, at first glance, it may be concluded that all these seven pillars have equal weight in the formation of friendships. But this conclusion is not true in general. Bahns *et al.* [27] identified the shared importance of attitude as highly significant. On the other hand, Galupo & Gonzalez [28] compared general friendship values and cross-identity values. From [27,28], it can be seen that some factors weigh more in comparison to others for friendship between two individuals. Since there are many factors behind the friendship of two individuals, we are confined to only seven pillars, as mentioned by Dunbar [17] in this paper.

Let (*F*, *A*) denote a soft set, where the set of attributes *A* contains the people under consideration and the universal set on which it is defined contains seven dimensions for the foundation of friendship as mentioned above. Also, let (*G*, *B*) be a soft set where the set *B* contains one human being, for which we have to check whether the rest of the people belonging to *A* may or may not have a chance to become friends. Then, we can calculate the possible deepness of friendship *D*(*a*, *b*) between two persons *a* ∈ *A* and *b* ∈ *B* by the following formula:

$$D(a,b)=\frac{\left|F(a)\cap G(b)\right|}{\max\{|F(a)|,|G(b)|\}},$$

where |*F*(*a*)| and |*G*(*b*)| denote the cardinalities of the sets *F*(*a*) and *G*(*b*), respectively. If *D*(*a*, *b*) = 1, then there is a full chance that *a* and *b* are good friends with each other and if *D*(*a*, *b*) = 0, then there is no chance that *a* and *b* are friends with each other. Here, |*F*(*a*)| and |*G*(*b*)| are at least 7 (because a person maps to at least one value of each kind of attribute in the universal set *X* through the mappings *F* and *G*). In fact, for a more detailed analysis of the deepness of friendship between two people, we have to partition the set *X* as finely as possible. Let us now take an example to illustrate the above formula.

**Example 3.1.**

Let *A* = {*a*_1_, *a*_2_, *a*_3_, *a*_4_, *a*_5_, *a*_6_, *a*_7_, *a*_8_, *a*_9_} and *B* = {*a*_10_} be two sets of persons. We now calculate *D*(*a*_2_, *a*_10_), i.e. the possible deepness level of friendship between *a*_2_ and *a*_10_, as indexed by the degree of homophily between the two individuals. Here, the universal set consists of languages, names of places around the world, educational histories, hobbies, world views, sense of humour and the musical taste of the people in sets *A* and *B*. Now, we define the soft set (*F*, *A*) as follows: *F*(*a*_1_) = {English, New York, bachelor of science from Oxford University, singing, likes politics, has a good sense of humour, rock music}, *F*(*a*_2_) = {English, London, master of arts from Oxford University, playing cricket, hates politics, has a bad sense of humour, folk music}, and so on. Similarly, the soft set (*G*, *B*) is defined as *G*(*a*_10_) = {English, London, master of arts from Oxford University, playing cricket, likes politics, has a bad sense of humour, rock music}. Hence, $|F(a_{2})\cap G(a_{10})|=5$ and *D*(*a*_2_, *a*_10_) = 5/7 = 0.71428. From this analysis, we can conclude that if persons *a*_2_ and *a*_10_ are friends, then they have more than average deepness of friendship. Here, we can make many finer partitions of the sets *F*(*a*_2_) and *G*(*a*_10_) to obtain more efficient results.

**Theorem 3.2.**

*If* *D*(*a*, *b*) *denotes the level of deepness of friendship between two persons* *a* *and* *b*, *then the following properties hold*:

(i)
0 ≤ *D*(*a*, *b*) ≤ 1,
(ii)
*D*(*a*, *b*) = *D*(*b*, *a*),
(iii)
*if* *D*(*a*, *b*) > 0.5 *and* *D*(*b*, *c*) > 0.5, *then* *D*(*a*, *c*) > 0.

**Proof.**

We only prove (iii).

**Figure 2.**
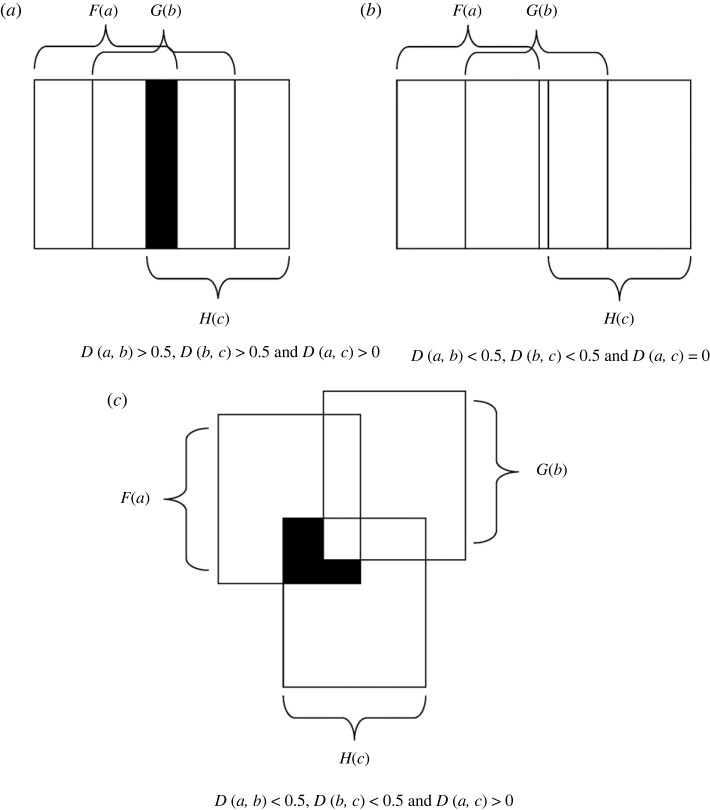
(*a*) Friendship between two persons *a* and *c* exists if the deepness of friendship between persons *a* and *b* is greater than 0.5 and the deepness of friendship between persons *b* and *c* is greater than 0.5; (*b*) possibility of non-existence of friendship between two persons *a* and *c* if the deepness of friendship between persons *a* and *b* is less than 0.5 and the deepness of friendship between persons *b* and *c* is less than 0.5; (*c*) possibility of existence of friendship between two persons *a* and *c* exists if the deepness of friendship between persons *a* and *b* is less than 0.5 and the deepness of friendship between persons *b* and *c* is less than 0.5.

Let (*F*, *A*) and (*G*, *B*) be two soft sets such that *a* ∈ *A* and *b* ∈ *B* as discussed above. We have, *D*(*a*, *b*) > 0.5, i.e. *G*(*b*) covers more than half of the set *F*(*a*), where *F*(*a*) ∈ *τ*{(*F*, *A*)} and *G*(*b*) ∈ *τ*{(*G*, *B*)}. Again, let (*H*, *C*) be another soft set such that *c* ∈ *C*. Then, *D*(*b*, *c*) > 0.5 implies *H*(*c*) covers more than half of the set *G*(*b*), where *G*(*b*) ∈ *τ*{(*G*, *B*)} and *H*(*c*) ∈ *τ*{(*H*, *C*)}. Hence, F(a)∩G(b)∩H(c)≠∅ as shown in figure 2 *a*. The black portion in the figure denotes the set F(a)∩G(b)∩H(c) . From this we can conclude that F(a)∩H(c)≠∅ , i.e. |F(a)∩H(c)|>0 . Thus, D(a,c)=|F(a)∩H(c)|/max{|F(a)|,|H(c)|}>0 .■

Now, let us analyse three situations as shown in the figure 2. For this, consider the following example:

**Example 3.3.**

Let *A* = {*a*}, *B* = {*b*}, and *C* = {*c*} be the sets of three persons. We form three soft sets (*F*, *A*), (*G*, *B*) and (*H*, *C*) over the universal set *X*, which contains the elements representing the seven pillars of friendship. The three soft sets are given by: *F*(*a*) = {language 1, language 2, language 3, school 1, college 1, university 1, hobby 1, hobby 2, hobby 3, has good sense of humour, moral view 1, moral view 2, religious view 1, political view 2}, *G*(*b*) = {language 1, language 2, language 4, language 5, school 2, college 1, university 2, hobby 2, hobby 3, hobby 7, has good sense of humour, moral view 2, moral view 3, religious view 1, political view 1}, and *H*(*c*) = {language 1, language 2, language 5, school 1, college 2, university 1, hobby 1, hobby 2, hobby 3, has good sense of humour, moral view 1, religious view 1, religious view 2, political view 1, political view 2}. Hence, we have *D*(*a*, *b*) = 8/15 = 0.533, *D*(*b*, *c*) = 8/15 = 0.533, and *D*(*a*, *c*) = 11/15 = 0.733 > 0, i.e. if *D*(*a*, *b*) > 0.5 and *D*(*b*, *c*) > 0.5, then, *D*(*a*, *c*) > 0. Thus, there is a chance of friendship between *a* and *c* as *D*(*a*, *c*) > 0, which is described in figure 2 *a*.

Now, we consider figure 2 *b*. For this, we define the soft sets (*F*, *A*), (*G*, *B*), and (*H*, *C*) as follows: *F*(*a*) = {language 1, language 2, language 3, school 1, college 1, university 1, hobby 1, hobby 2, hobby 3, has good sense of humour, moral view 1, moral view 2, religious view 1, political view 2}, *G*(*b*) = {language 1, language 2, language 3, school 2, college 2, university 2, hobby 1, hobby 4, hobby 5, has good sense of humour, moral view 2, moral view 3, religious view 2, political view 4}, and *H*(*c*) = {language 4, language 5, language 6, school 2, college 2, university 3, hobby 4, hobby 5, hobby 6, has bad sense of humour, moral view 3, moral view 5, religious view 3, political view 5}. Now, *D*(*a*, *b*) = 6/14 = 0.428, *D*(*b*, *c*) = 5/14 = 0.357, and *D*(*a*, *c*) = 0/14 = 0, which is described in figure 2 *b*, i.e., if *D*(*a*, *b*) < 0.5 and *D*(*b*, *c*) < 0.5, then there is a possibility that *D*(*a*, *c*) = 0.

At last, we consider figure 2 *c*. For this, we define the soft sets (*F*, *A*), (*G*, *B*), and (*H*, *C*) as follows: *F*(*a*) = {language 1, language 2, language 3, school 1, college 1, university 1, hobby 1, hobby 2, hobby 3, has good sense of humour, moral view 1, moral view 2, religious view 1, political view 2}, *G*(*b*) = {language 1, language 2, language 3, school 2, college 2, university 2, hobby 1, hobby 4, hobby 5, has good sense of humour, moral view 2, moral view 3, religious view 2, political view 4} and *H*(*c*) = {language 4, language 5, language 6, school 2, college 2, university 3, hobby 4, hobby 5, hobby 6, has bad sense of humour, moral view 3, moral view 5, religious view 3, political view 2}. Now, *D*(*a*, *b*) = 6/14 = 0.428, *D*(*b*, *c*) = 5/14 = 0.357 and *D*(*a*, *c*) = 1/14 = 0.071, i.e. if *D*(*a*, *b*) < 0.5 and *D*(*b*, *c*) < 0.5, then there is a possibility that *D*(*a*, *c*) > 0.

In the above example, *a*, *b* and *c* indicate that there are three individuals present. In this case, friends *a* and *b* have a below-average degree of closeness. Persons *b* and *c* are also friends, but their friendship is less close-knit than usual. Therefore, based on these two connections, it is impossible to accurately forecast the level of friendship between *a* and *c*. However, according to theorem 3.1, for *a* and *c* to be friends, they must share at least a few things in common if *a* and *b* and *b* and *c* have powerful, deep friendships.

### 3.1. Effect of romantic relationship on friendship

Burton *et al.* [1] mentioned that the cost of romance is the loss of nearly two members from an individual’s support network when the individual begins a new relationship. The reason is due to the time investment for a friend or the romantic relationship. In [17], we get to know that the closest support network consists of five people on average. This situation can be analysed mathematically by defining a time function *T*. So, we have the following definition:

**Definition 3.4.**

Let *A* = {*a*_1_, *a*_2_, *a*_3_, *a*_4_, *a*_5_} be the set of five persons that forms the support network of *X*. Then, we define a function *T* such that T : {X}×A⟶[0,1] by $T(X,a_{i})=t_{X,a_{i}}$ , where $t_{X,a_{i}}\in [0,1]$ indicates the time spend by *X* with *a*_*i*_. Here, we assume $\sum_{i=1}^{5}T(X,a_{i})=1$ .

Since the average number of people in one’s closest support network is five, which we consider to be the cardinality of the set *A*, one can change the cardinality of *A* by giving it a slightly different value. For convenience, we are using the average. Now, let us define the weight of a friendship (W) as follows:

**Definition 3.5.**

The weight of friendship between two persons *a* and *b*, denoted by *W*(*a*, *b*), is defined by *W*(*a*, *b*) = *D*(*a*, *b*) × *T*(*a*, *b*).

From the above definition, we can see that the more time a person spends with another person, the greater their weight of friendship. Also, it is obvious that 0 ≤ *W*(*a*, *b*) ≤ 1. Now, we define some mathematical results related to weight-after and weight-before.

Let a person *X* have a supporting network of a set of five persons {*a*_1_, *a*_2_, *a*_3_, *a*_4_, *a*_5_}. Suppose *X* is getting into a new relationship with an individual *p*. Without loss of generality, we will study the change in weight of friendship between *X* and *a*_*i*_, *i* ∈ {1, 2, 3, 4, 5}. The weight of friendship before *X* gets into a relationship is given by

$$W_{\mathrm{before}}(X,a_{i})=D(X,a_{i})\times \frac{\sum_{i=1}^{5}T(X,a_{i})}{5}.$$

The weight of friendship after *X* gets into a relationship is given by

$$W_{\mathrm{after}}(X,a_{i})=D(X,a_{i})\times \frac{\left(\sum_{i=1}^{5}T(X,a_{i}))-T(X,p\right)}{5}.$$

Thus,

$$\frac{W_{\mathrm{before}}(X,a_{i})}{W_{\mathrm{after}}(X,a_{i})}=\frac{\sum_{i=1}^{5}T(X,a_{i})}{\left(\sum_{i=1}^{5}T(X,a_{i}))-T(X,p\right)}=\frac{1}{1-T(X,p)}.$$

Hence,

$$W_{\mathrm{after}}(X,a_{i})=(1-T(X,p))W_{\mathrm{before}}(X,a_{i}).$$

From the above discussion, we conclude the following theorem:

**Theorem 3.6.**

*If two persons* *X* *and* *a*_*i*_ *are friends and* *X* *is getting into a new relationship with* *p*, *then the change in weight of friendship between* *X* *and* *a*_*i*_ *can be determined by the following limits*:

(i)
*if* T(X,p)⟶1 , *then* $W_{\mathrm{after}}(X,a_{i})⟶0$ ,
(ii)
*if* T(X,p)⟶0 , *then* $W_{\mathrm{after}}(X,a_{i})⟶W_{\mathrm{before}}(X,a_{i})$ , *i* = 1, 2, 3, 4, 5.

**Proof.**

(i)

$$\begin{array}{rl} & \lim_{T(X,p)\to 1}W_{\mathrm{after}}(X,a_{i})\\ & \;=\lim_{T(X,p)\to 1}\{(1-T(X,p))W_{\mathrm{before}}(X,a_{i})\}\\ & \;=\lim_{T(X,p)\to 1}\{(1-T(X,p))\}\lim_{T(X,p)\to 1}\{W_{\mathrm{before}}(X,a_{i})\}\\ & \;=0.\;W_{\mathrm{before}}(X,a_{i})=0. \end{array}$$

(ii)

$$\begin{array}{rl} & \lim_{T(X,p)\to 0}W_{\mathrm{after}}(X,a_{i})\\ & \;=\lim_{T(X,p)\to 0}\{(1-T(X,p))W_{\mathrm{before}}(X,a_{i})\}\\ & \;=\lim_{T(X,p)\to 0}\{(1-T(X,p))\}\lim_{T(X,p)\to 0}\{W_{\mathrm{before}}(X,a_{i})\}\\ & \;=1.W_{\mathrm{before}}(X,a_{i})=W_{\mathrm{before}}(X,a_{i}). \end{array}$$

■

**Figure 3.**
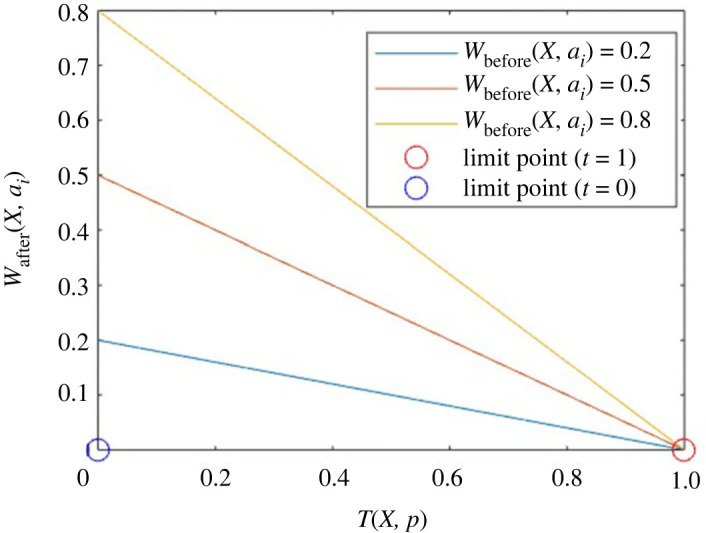
Relation between *W*_after_ and *W*_before_ with time *T*(*X*, *p*).

Let us conduct a visual analysis of the problem. We take into account three instances in figure 3. Before entering a relationship, a person has a weight of 0.8 with one of their friends. Gradually, when he begins to spend more time with the romantic partner after entering a relationship, the weight of friendship with the friend fades away. Similar circumstances arise if the person has two additional friends with friendship weights of 0.5 and 0.2, respectively. Additionally, it is clear from figure 3 that even though a person who has a high weight of friendship with friends begins to spend more time with his romantic partner, he will still have a higher weight of friendship with the friend than someone who has a low weight of friendship before a relationship.

Burton *et al.* [1] mentioned that we lose two good friends after getting into a romantic relationship. That is, on average, almost 40% of the time we spend with our romantic partner. Thus, mathematically, if T(X,p)⟶0.4 then $W_{\mathrm{after}}(X,a_{i})⟶W_{\mathrm{before}}(X,a_{i})\times 0.6$ .

**Definition 3.7.**

Let W* denote the total weight of friendship of five persons {*a*_1_, *a*_2_, *a*_3_, *a*_4_, *a*_5_} with another person *X* before entering into a romantic relationship. Then, W* is given by: $W^{*}=\sum_{i=1}^{5}W(X,a_{i})$ . If *W* ^**^ denotes the total weight of friendship of five persons {*a*_1_, *a*_2_, *a*_3_, *a*_4_, *a*_5_} with another person *X* after he enters into a romantic relationship with *p*, then *W* ^**^ is given by: $W^{**}=\sum_{i=1}^{5}W(X,a_{i})-W(X,p)$ .

**Theorem 3.8.**

*The supremum of* *W* ^**^ *is* {1 − 0.08 × *D*(*X*, *p*)}W*.

**Proof.**

From the above definition, $W^{**}/W^{*}=1-(W(X,p)/\sum_{i=1}^{5}W(X,a_{i}))=1-(D(X,p)\times T(x,p)/\sum_{i=1}^{5}[D(X,a_{i})\times T(x,a_{i})])$

Now, we have $\sum_{i=1}^{5}W(X,a_{i})\leq 5$ and we also know that T(X,p)⟶0.4 . Thus,

$$\begin{array}{rl} \frac{W^{**}}{W^{*}} & =1-\frac{W(X,p)}{\sum_{i=1}^{5}W(X,a_{i})}=1-\frac{D(X,p)\times T(x,p)}{\sum_{i=1}^{5}[D(X,a_{i})\times T(x,a_{i})]}\\ & \leq 1-\frac{D(X,p)\times 0.4}{5}=1-\{0.08\times D(X,p)\} \end{array}$$

Thus, *W* ^**^ ≤ {1 − (0.08 × *D*(*X*, *p*)}W*. Hence, the theorem is proved.■

From this inequality, we can predict the overall decrease in the weight of friendship among the five good friends after a person enters a new romantic relationship.

### 3.2. Balance theoretic analysis of friendship through soft set approach

The quantity ‘deepness of friendship’ defined above has an important role in the formation of friendships, but it doesn’t guarantee that having a high deepness will represent a closer friendship. It only indicates that if friendship happens between two people, then their bonding will be stable if they have a fair deepness of friendship. The reason is that the quantity *D*(*a*, *b*) is based on the seven pillars of friendship [17], and two individuals having all the elements in common in the seven pillars don’t yet know each other, so there is no friendship between them. But in the case of the quantity ‘weight of friendship’, it also includes the time function, hence, we conclude that if two individuals having a fair deepness of friendship between them spend some time with each other, then there is a fair weight of friendship between them, and thus we say they are friends. Therefore, in this section, we will use the weight of friendship along with balance theory [29] to analyse the scenario of the formation of Dunbar’s hierarchy layers for an individual and calculate the number of ‘cracks’ or ‘weak bonding’ a person can tolerate until the personal social network becomes unstable. For this, let us introduce some preliminaries regarding the balance theory [29].

Heider first presented the balance theory [29] as a justification for attitude shift, then Cartwright and Harary explicitly generalized it for graphs [30]. Balance theory provides a tool for measuring how balanced or stable a relationship is. The original definition solely considered whether or not graphs were balanced; by ‘balanced’, it was understood that all cycles among all nodes contained exactly an even number of negative edges [30]. Later research claimed that the majority of social science applications of balance theory only applied to triads of nodes, or connected triples [31]. Here, we also consider the concept of balanced triples for constructing the social support network of an individual. Balanced configurations are still those with an even number of negative edges. In particular, we define the concepts of ‘The friend of my friend is also my friend’ [ + + + ] and ‘The enemy of my friend is my enemy’ [ + − − ] as balanced or stable. The two additional varieties of signed triad configurations, [ + + − ] and [ − − − ], are regarded as unstable and cause network fractiousness. As a further refinement, social scientists since [32,33] have observed that the two types of balanced triads are not equally balanced, and the two types of frustration are not equally frustrated. The state [ + + + ] is more stable than the state [ + − − ]. In this case, we are not going to deal with unstable triads and the state [ + − − ] because our goal is to establish a stable friend network for an individual rather than identify their enemy network.

Now, before constructing an algorithm for the formation of a stable support network for an individual we mention some of the interesting results about friendship. The amount of time devoted to a particular social relationship strongly affects the emotional quality of a friendship [34]. According to one prospective study, it takes over 200 hours of face-to-face interaction spread out over a three-month period to make a good friend [35]. However, time is naturally scarce; we only spend roughly 20% of each day engaging in direct social interaction (excluding those related to work), or 3.5 hours [36]. Given that not all of our relationships are equally valuable to us, we divide up our precious time among our social network to make the most of the various advantages that friends of various calibres may offer [37]. However, there are some patterns that are largely constant: we spend 40% of our time with our closest friends and family, and another 20% with the next 10 closest people. In other words, only 15 people receive 60% of the 3.5 hours per day that we spend interacting with others. Only 30 seconds per day, on average, are spent with social partners in the social network’s outermost tiers. Our social networks, as a result, develop a very distinctive layering with levels that follow a specific fractal structure [36]. From this data, we can conclude that time spent with someone is a very important factor for a stable relationship with that individual. Let us calculate the value of the friendship function between two individuals for which one would belong at least among the 150 people of the other person’s Dunbar’s hierarchy.

**Definition 3.9.**

Let S : {p}×X⟶[0,86400] such that *S*(*p*, *x*) = *t* × *D*(*p*, *x*), where *t* ∈ [0, 86400], *D*(*p*, *x*) ∈ [0, 1] and *X* is the set of people that are known to the person *p*, and *t* denotes the time *p* spends with *x* in seconds in one day. Also, *D*(*p*, *x*) denotes the deepness of friendship between *p* and *x*. Here, we take the range of *S* as [0, 86400] because a day consists of 86400 seconds. The function *S* is called the friendship function for *p* and *x* ∈ *X*.

Now, let us calculate the smallest value of the friendship function for which a person *x* resides at least in the outermost layer of Dunbar’s hierarchy of the individual *p*. Since only 30 seconds per day, on average, are spent with social partners in the social network’s outermost tiers of an individual, we get *S*(*p*, *x*) = 30 × *D*(*p*, *x*). The value of *D*(*p*, *x*) is subject to the individual *p*; hence, we have not assigned any particular value to it. For now, we consider this value a threshold for *x* to reside at least at the outermost layer of the social network structure of individual *p*.

### 3.3. Stability of Dunbar’s hierarchy layers from the perspective of balance theory

**Figure 4.**
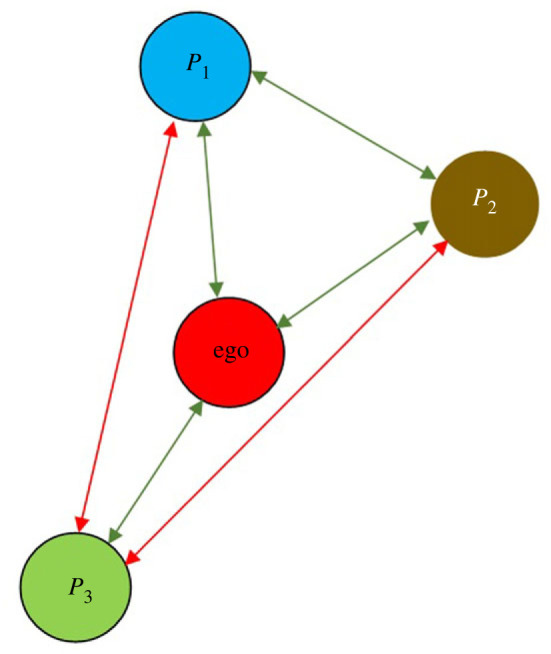
Graphical representation of the algorithm to construct a stable support network for a person *P*_0_.

In this section, we consider the balanced triad [ + + + ] only. As shown in figure 4, let the ‘ego’ reside in the middle, and their Dunbar’s hierarchy consists of persons *p*_1_, *p*_2_, *p*_3_, and so on. We denote stable relations by the green lines and unstable relations by the red lines in the figure. Now we follow the following algorithm to develop the layers of Dunbar’s hierarchy for the ‘ego’. Before proceeding with the steps, we assume a hypothesis. We are taking this hypothesis because, according to a model developed by Conradt & Roper [38], in situations involving conflicting interests, decisions made by the majority of the group should be advantageous because they avoid extreme outcomes by averaging over individual preferences, keeping the consensus costs reasonable for each person. Also, in [39], Dyer *et al*. provided support for the model of Couzin *et al.* [40] showing that where differences in preference are large and there is an imbalance in the number of individuals with each directional preference, human groups tend to choose the direction preferred by the majority.

**Hypothesis.**

*If there are* *n* *persons in a network of an individual, where* *n* *is an odd number, then the* (*n* + 1)^th^ *person can enter the network if at least* ⌈n/2⌉ *persons that already exist in the network have a good relationship with the* (*n* + 1)^th^ *person, and if* *n* *is an even number, then the* (*n* + 1)^th^ *person can enter the network if at least* ⌈(n/2)+1⌉ *persons that already exist in the network, have a good relationship with the* (*n* + 1)^th^ *person to have a stable network. Here*, ⌈n⌉ *denotes the standard ceiling function*.

This hypothesis is totally subjective, i.e. it totally depends on the ‘ego’ that we consider. The least value of the persons ( i.e. ⌈n/2⌉ or ⌈(n/2)+1⌉ ) may differ for different ‘ego’. Next, we move to the following steps:

1.
Construct a set *A* of individuals in ascending order that have friendship functions with values above the threshold, where the ‘ego’ is concerned. Thus, let *A* = {*p*_1_, *p*_2_, …, *p*_*n*_}.
2.
According to our hypothesis, *p*_1_ will enter the social network of the ‘ego’ as shown in figure 4, because *p*_1_ has a positive relationship with the ego, and similarly, *p*_2_ will also enter the network because *p*_2_also has a positive relationship with both the ego and *p*_1_. However, *p*_3_ will not join the network because *p*_3_ has two negative relationships with the other existing members of the network.
3.
We continue the process until there are too many weak bonds between the members of Dunbar’s hierarchy layers of the ‘ego’ so that no more tolerance can be imposed on her.
4.
The people with fewer weak bonds will be placed in the inner layers compared to those with more weak bonds in Dunbar’s hierarchy.

**Definition 3.10.**

Let there be *n* people in the support network of an individual *p*. If a new person enters the network of *p* and has bad relations with *m* people that already exist in the network of *p*, then we call the number *m* as *m* cracks or *m* weak bonds in the network.

**Theorem 3.11.**

*If there are* *n* *people in the social network of an individual* *p*, *then the maximum number of cracks or weak bonds in the network that can be tolerated by*

$$p=\{\begin{array}{cc} \frac{n^{2}-2n+1}{4}, & \text{i}f\;n\;\text{i}s\;\text{o}dd,\\ \frac{n^{2}-2n}{4}, & \text{i}f\;n\;\text{i}s\;\text{e}ven. \end{array}$$

**Proof.**

We calculate the maximum number of cracks according to our hypothesis and algorithm when the first individual will start to come to the network of *p* from the set *A*. We consider the following two cases:

*Case 1:* When *n* is odd, $\text{then total number of cracks}=1-\lceil 1/2\rceil +2-\lceil (2/2)+1\rceil +3-\lceil 3/2\rceil +4-\lceil (4/2)+1\rceil +5-\lceil 5/2\rceil +6-\lceil (6/2)+1\rceil +7-\lceil 7/2\rceil +\cdots +n-\lceil n/2\rceil =(n^{2}-2n+1)/4$ .

*Case 2:* When *n* is even, $\text{then total number of cracks}=1-\lceil 1/2\rceil +2-\lceil (2/2)+1\rceil +3-\lceil 3/2\rceil +4-\lceil (4/2)+1\rceil +5-\lceil 5/2\rceil +6-\lceil (6/2)+1\rceil +7-\lceil 7/2\rceil +\cdots +n-\lceil (n/2)+1\rceil =(n^{2}-2n)/4$ .■

**Corollary 3.12.**

*For Dunbar’s number* *n* = 150, *the maximum number of cracks or weak bonds that can occur in the stable network of an individual is 5550. Hence, the minimum number of stable or strong bonds that is required in Dunbar’s hierarchy of an individual is* ^*n*^ *C*_2_ − 5550 = ^150^ *C*_2_ − 5550 = 5625. *Thus, based on balance theory, at least 5625 interpersonal strong bonds are required among the individuals to have one’s social networks with* *n* = 150 *persons stable or balanced*.

According to our algorithm, an ego should have at least minimal interactions with the people that reside inside his Dunbar’s hierarchy. But it is not at all possible to interact with all the people under consideration when time is limited. Hence, after a certain number of friends in our networks, we start to get people who are uncommitted and have little attachment to the ego. Since 60 percent of the time of an ego is spent with the first 15 people in their Dunbar hierarchy, after that, we are left with only 40 percent of 3.5 hours (i.e., 5040 seconds) for the remaining people in the network. For ease of calculations and because humans are rational [41], we assume that the ego divides the remaining time equally among their friends so that we can get an approximate number of friends who have a little attachment to the ego based on time spent. Since only 30 seconds, on average, are spent by an ego with the people in the outermost layers, we get approximately 168 people with whom they spent at least 30 seconds. Hence, the total number of people with at least a little personal interaction with the ego is 183.

Therefore, if the population of the ego’s network consists of *n* people, then *n* > 183, in order to calculate the probability that we have discovered an uncommitted person with whom one does not spend time. Consequently, the probability of seeing an ambivalent person in one’s Dunbar hierarchy is 1 − (183/*n*).

### 3.4. Marriage, divorce and Dunbar’s hierarchy

According to Afifi *et al.* [42], cultures influence the processes of marriages and divorces. Our society is what gives us our culture, and the members of our society are the ones we deal with on a daily basis. As a result, those who are higher up in Dunbar’s hierarchy of an individual also have an impact on that person in these areas. More generally, if a society is used to divorce, then no one in that society would hesitate to divorce their partner, demonstrating that the process is at least in part influenced by the culture created by the individuals in one’s society. [43]. Our theory holds that when a male marries a female, the female will naturally reside in the core layer of the male in Dunbar’s hierarchy. Because of emotional attachments, supporting nature to her husband and his family, intimacy, etc., the marriage may indicate an increase in the male’s hierarchy with those who are coming from the bride’s side and a decrease in the hierarchy with those who already existed in some outer layers of the male’s hierarchy. However, the outcome of a divorce is the exact opposite. Additionally, the stability of any male’s or female’s network may be compromised by the loss of their former spouse if they find someone better or more comparable to them in the sense of the deepness and weight indicated above.

**Figure 5.**
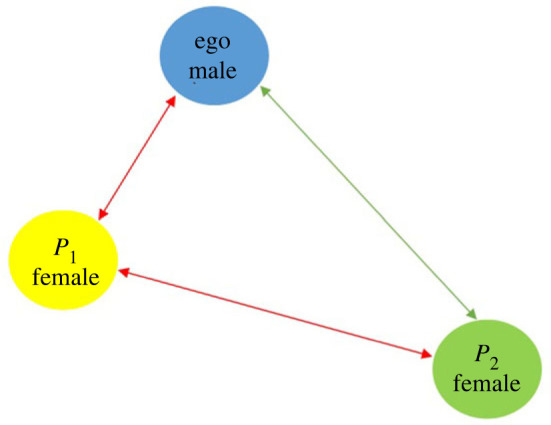
Representation of divorce in an imbalanced triad.

But the reason for divorce is very different from the seven pillars that we have discussed and the time factor that we introduced. Apostolou *et al.* [44] used qualitative research techniques to identify 62 probable causes of divorce. Through the use of quantitative research techniques, they categorized these causes into seven general components. The ability to have children and financial difficulties were found to be the least important factors. They discovered that being a harmful spouse—having extramarital relationships, being abusive, being addicted to gambling or drugs, etc—were the most important factors that could lead people to divorce, followed by incompatibility and in-law problems. Hawkins *et al.* [45] also discussed several reasons for divorce. As seen in figure 5, when the ego feels better with partner *P*_2_ than with partner *P*_1_ due to the aforementioned issues, there will be an imbalance triad in the network because, in this scenario, both *P*_1_ and *P*_2_ would be located in the ego’s hierarchy and have both positive and negative relations with him. Therefore, they choose to make the triad balance as [ − − + ] in order to keep it stable, and in doing so, the ego may seek to divorce his previous partner *P*_1_. As a result, we may draw the conclusion that, in order to analyse the stability and instability of a personal network system during divorce, functions must be defined in terms of the variables that affect the process.

From the perspective of a divorce, we are attempting to determine the possibility that a person will have an extramarital affair. Extramarital affairs have a significant impact on divorce [44]. Therefore, when a marriage dissolves due to extramarital activities, we assume that the person with whom the ego has an extramarital relationship is hated by his or her former spouse and that they have a weak or unfavourable bond while forming a triad with the ego. Instead of analysing the likely causes of divorce, we look at the likelihood of an extramarital affair that causes the divorce from the perspective of a personal network system based on the balance theory. Let there be *m* − 1 persons in the personal network of an individual *p* while he gets married to *p*_1_. Hence, according to our algorithm, there are some cracks that are already in the network, and while the spouse enters the network, the number of cracks increases or remains the same. After the succession of the marriage, we assume *n* people have entered the network, and we take the new partner *p*_2_, with whom the ego has an extramarital affair, as the *n*th person in the network. Now, we need to know how many weak bonds there are in the network between the time the marriage is over and the *n*th person enters it. It is due to the fact that one of these cracks will represent the weak relationship between *p*_1_ and *p*_2_. Now, to find the possibility that *p*_2_ is having an extramarital affair with *p*, we have the following four cases:

*Case 1:* When *m* + *n* is odd and *m* is also odd, then the minimum likelihood that *p* has an extramarital affair with *p*_2_ = 1/((((*m* + *n*)^2^ − 2(*m* + *n*) + 1)/4) − ((*m* ^2^ − 2*m* + 1)/4) = 4/(*n* ^2^ + 2*mn* − 2*n*)).

*Case 2:* When *m* + *n* is odd and *m* is even, then the minimum likelihood that *p* has an extramarital affair with *p*_2_ = 1/((((*m* + *n*)^2^ − 2(*m* + *n*) + 1)/4) − ((*m* ^2^ − 2*m*)/4) = 4/(*n* ^2^ + 2*mn* − 2*n* + 1)).

*Case 3:* When *m* + *n* is even and *m* is odd, then the minimum likelihood that *p* has an extramarital affair with *p*_2_ = 1/((((*m* + *n*)^2^ − 2(*m* + *n*))/4) − ((*m* ^2^ − 2*m* + 1)/4) = 4/(*n* ^2^ + 2*mn* − 2*n* − 1)).

*Case 4:* When *m* + *n* is even and *m* is also even, then the minimum likelihood that *p* has an extramarital affair with *p*_2_ = 1/((((*m* + *n*)^2^ − 2(*m* + *n*))/4) − ((*m* ^2^ − 2*m*)/4) = 4/(*n* ^2^ + 2*mn* − 2*n*)).

If a male finds someone better than his former partner, then we assume that the person is coming to the Dunbar’s hierarchy of the male after his meeting with the former partner. So, we should consider the individuals who enter Dunbar’s hierarchy of a male either after he marries or after he first meets his former partner in order to determine the possibility that he will discover someone better than his former partner. Since the male always has a positive relationship with the people in his Dunbar’s hierarchy, in this case, if the first partner of the male is the *n*th person in the hierarchy, and there are a total of *m* people in the network at a specific time while the male finds someone better than his former partner, then the probability that a person from the list of the *m* − *n* people that enters the hierarchy after the male meets his second partner is better than his former partner is 1/(*m* − *n*). But if the person with whom the male feels better after marriage existed in his network before the marriage, then the probability will be 1/(*m* − 1). The same result can be obtained for a female.

## 4. Conclusion

The most crucial elements that affect a person’s mental health are friendship and relationships [46–48]. In §2, we can see how getting married changes the conjoint Dunbar graph and a person’s relationship with another person through some concepts of soft narrow unions and soft narrow intersections. On the other side, we use soft set theory to gauge the strength of friendship between two people, which is dependent on the seven factors [17] that Dunbar listed. Additionally, we determined the importance of friendships between two people, which is based on both the amount of time spent with friends and the seven criteria. The similarities between these concepts and actual world circumstances have been graphically illustrated. Based on Dunbar’s approximation [17], we have also found the supremum value of a person’s overall weight of friendship under the influence of relationship. We next procure the balance theory to get the findings about the stability of a person’s social network. We developed an algorithm to determine how large a person’s social network can be so that it stays stable. We also looked at the marriage and divorce scenario and determined the likelihood of extramarital affairs.

We found that the seven pillars of friendship [17] do not weigh the same. There are several other factors responsible for friendship between two people [24–26]. Some factors weigh more in comparison to others. In this paper, we confined ourselves only to the seven pillars of friendship [17]. From [17], we know that the closest support network consists of five people on average. Although uniform numbers of friends can be found in this case, this is a skewed distribution. This skewed distribution does not affect our results in any way. For example, if we assume three members in one’s support network, then the equality *W*_after_(*X*, *a*_*i*_) = (1 − *T*(*X*, *p*))*W*_before_(*X*, *a*_*i*_) is still consistent, except for the alternation of the upper limit of the summation for *i* as 3 in lieu of 5. In this case, *W*_before_(*X*, *a*_*i*_) for *i* = 1, 2, 3 will be more because the person will get more time for friends in comparison to *W*_before_(*X*, *a*_*i*_) for *i* = 1, 2, 3, 4, 5. Similarly, the person will also spend more time with a romantic partner. It yields a higher value of *T*(*X*, *p*). But the figure in this case, after simulation, will also be similar, as shown in figure 3. In short, we will get a family of straight lines altering the values of *W*_before_(*X*, *a*_*i*_) as shown in figure 3.

The uses of soft set theory help to provide better models of relationships in comparison to others because soft set theory connects attributes and related approximations [18,19,21]. We found that both friendship and relationship are connected to several attributes; hence, we defined the deepness of friendship *D*(*a*, *b*) between two persons *a* and *b* using soft set theory. The definition 3.2 uses *D*(*a*, *b*) to define the weight of friendship *W*(*a*, *b*) between two persons *a* and *b*. In general, a person spends more time with a romantic partner compared to a new friend entering the support network [49–51]. Moreover, the person becomes more emotionally or physically attached to the romantic partner or trustworthy of the romantic partner as time passes in comparison to other friends [49–51]. There are several other factors that differentiate a romantic partner from other friends [52]. It may be possible that a new friend will become a romantic partner. In that case, the results of §3.1 will be valid. For one’s extramarital affair or new relationship in the presence of a previous romantic partner, §3.4 provides a suitable description. Moreover, theorem 3.4 helps us study marriage, divorce, and Dunbar’s hierarchy. It tells us about the stability of one’s supporting network depending on the position of the romantic partner by using balanced triads. We found that a minimum of 5625 stable or strong bonds are required for the stability of one’s social network of 150 people. It means that a minimum of ^5625^ *C*_3_ = 29 647 267 500 balanced triads of [ + + + ] are required for the stability of one’s social network of 150 people. Thus, the above results help balance theory to find the stability of one’s social network. Since one has the scope to associate different weights with factors of friendship, one may explore new theories regarding this, as motivated by this paper. In the near future, we anticipate that these mathematical concepts will be useful in analysing human behaviour regarding friendship, relationship, etc. from the perspective of human-computer interactions and artificial intelligence.

## Data Availability

This article has no additional data.
